# Translating principles of quality control to cardiovascular magnetic resonance: assessing quantitative parameters of the left ventricle in a large cohort

**DOI:** 10.1038/s41598-023-29028-7

**Published:** 2023-02-07

**Authors:** Leili Riazy, Sascha Däuber, Steffen Lange, Darian Steven Viezzer, Steffen Ott, Stephanie Wiesemann, Edyta Blaszczyk, Fabian Mühlberg, Leonora Zange, Jeanette Schulz-Menger

**Affiliations:** 1 Experimental and Clinical Research Center (ECRC), Charité Universitätsmedizin Berlin-Buch, Berlin, Germany; 2 DZHK (German Centre for Cardiovascular Research), Partner Site Berlin, Berlin, Germany; 3 Siemens Healthineers, Erlangen, Germany; 4 Department of Computer Science, Darmstadt University of Applied Sciences, Darmstadt, Germany; 5 HELIOS Hospital Berlin-Buch, Berlin, Germany

**Keywords:** Data mining, Data processing, Quality control, Statistical methods, Magnetic resonance imaging, Medical imaging

## Abstract

Cardiac magnetic resonance (CMR) examinations require standardization to achieve reproducible results. Therefore, quality control as known as in other industries such as in-vitro diagnostics, could be of essential value. One such method is the statistical detection of long-time drifts of clinically relevant measurements. Starting in 2010, reports from all CMR examinations of a high-volume center were stored in a hospital information system. Quantitative parameters of the left ventricle were analyzed over time with moving averages of different window sizes. Influencing factors on the acquisition and on the downstream analysis were captured. 26,902 patient examinations were exported from the clinical information system. The moving median was compared to predefined tolerance ranges, which revealed an overall of 50 potential quality relevant changes (“alerts”) in SV, EDV and LVM. Potential causes such as change of staff, scanner relocation and software changes were found not to be causal of the alerts. No other influencing factors were identified retrospectively. Statistical quality assurance systems based on moving average control charts may provide an important step towards reliability of quantitative CMR. A prospective evaluation is needed for the effective root cause analysis of quality relevant alerts.

## Introduction

Cardiovascular Magnetic Resonance (CMR) is a valuable diagnostic tool increasingly used in clinical routine. It is generally regarded as the gold standard for quantification of the left ventricle^1,2^ such as the ejection fraction (EF), stroke volume (SV), end-diastolic volume (EDV), end-systolic volume (ESV) and left ventricular mass (LVM)^3^. CMR has unique capabilities to differentiate myocardial tissue with prevalent inflammation as well as ischemic injury. The differentiation of cardiomyopathies enables an advanced level of diagnostic quality^4–6^. The use of CMR is integrated in different clinical guidelines^2^ and changed the diagnostic pathways, i.e., in heart failure, specific diseases like myocarditis, ischemic disorders and/or left ventricular hypertrophy^7^.

As the method is less dependent on the operator compared to echocardiography and is characterized by a high repeatability and precision of the derived quantitative parameters, it provides several advantages. One example is the ability to significantly reduce the sample size in clinical trials^8,9^. However, the use of quantitative markers suffers from the lack of standardization. Consequently, parameters are not necessarily reproducible or acquired in a consistent way. The results can be influenced by a multitude of potentially unknown factors, both technical and human in nature. Therefore, a systematic quality assurance system for clinical settings is an essential step forward and is experiencing growing attention, especially as more and more Artificial Intelligence (AI)-generated quantitative parameters are entering the field^10^.

Quality in CMR imaging is fundamental as images and their derived parameters are used for therapy guidance. Therefore, the whole imaging chain has previously been a subject of intensive studies. Well established scores were used to objectify the quality of a given image^11^. However, this often stops with the assurance of image quality^12^ looking at the obvious dependencies from imaging sequences or protocols. However, beyond the image quality many other confounders can influence the whole imaging chain and lead to a degradation of the quantitative output, e.g., any human interaction or different analysis methods^13^. There are efforts from various societies to develop consensus criteria^14^ to mitigate the effects but the use is inconsistent and leads to different results^15^.

Two principal approaches of quality assurance are often followed^16^.

The first approach is to define a reference standard and use specific quality control material to check against that standard. This “snapshot” quality control is performed in regular intervals and on special occasions, e.g., before and after changes involving the analyzer. Comparable methods are applied in imaging by using a phantom or a specified set of volunteers^12,13^ but with severe limitations. A phantom does not fully represent the human body, using volunteers is not practical in clinical routine.

The second approach of quality assurance involves the analysis of the quantitative output itself, especially for the detection of long-term effects. Shewhart first introduced the control chart^17^, which has been adapted to clinical chemistry^16^. Specifically, for the detection of drifts in the data, the “Average of Normals” was introduced^18^. This procedure, often referred to as moving average or rolling mean/median, acts as a filter to remove natural variation in a group of measurements and can reveal drifts over time. This statistical quality control method has not been applied to imaging until now.

In this study, our aim is to prove that the method can detect unwanted deviations in measuring critical quantitative cardiac parameters that could potentially have adverse consequences when used for diagnostics or for taking therapy decisions.

## Materials and methods

In a high-volume CMR center, a new digital hospital information system was introduced in 2009. For CMR specific parameters, a software provided by SAP (Walldorf, Germany) has been used since 2010. The 5th of January 2010 defines the starting point of this analysis.

Quantitative CMR results are inserted into a predefined template containing all reports in a systematic manner. The clinical data management software allowed for an export of quantitative data, which was anonymized in order to avoid inclusion of any patient- or examiner-specific information. Due to the retrospective nature of our study, no written informed consent could be collected. The specific protocol of the present study has been approved by the ethical committee of Charité Universitätsmedizin Berlin during the instance EA1/253/21 and is therefore carried out in accordance with relevant guidelines and regulations. The exported dataset contained the following general fields: date of examination, age, weight, height, sex as well as the following LV function parameters: EF, LVM, SV, EDV. The LV ESV was not present in our data due to technical limitations, but was instead calculated as the difference of EDV and SV, as

$$ESV=EDV-SV.$$

The LV parameters were calculated using the commercial software cvi_42_^19^. Contours were drawn manually at the timepoint of reporting in accordance with specific operating procedures (SOP) and were supervised by an experienced reader. The contours were not edited thereafter. The SCMR guidelines^14^ were published within the timespan of this study. Although the contours partially predate the guideline publication, they conform to guideline standards. Throughout the whole time, the same MRI scanning protocol was used. The MRI scanner was upgraded in June 2014 and moved once in September 2016.

The Body Surface Area (BSA) is computed according to the formula given by Mosteller^20^.

The MRI scanner is dedicated to CMR only and approximately 2700 patient scans take place per year. The clinical range covers all indications with an underrepresentation of patients with congenital diseases, because the group focuses on acquired heart diseases^21^. All scans were performed on a 1.5 T Siemens MRI scanner and analyzed with various post-processing applications according to well-defined standard operating procedures. The software and hardware sub-systems experienced multiple updates. A well-defined training program allows for a high number of medical doctors to perform CMR. Every 6 months a new fellow joins the unit for an at least one-year fellowship to perform clinical routine CMR. The entries of new readers are documented. Highly trained experts (SCMR level 3) supervise the scans as well as the post-processing steps.

We are looking for confounding factors that are influencing the measurement chain and distort the LV function parameters. The confounding factors can include hardware and software changes, but there is also a human factor to be considered. Software changes include MR-sequences as well as different post-processing tools^13^ for image analysis. Human factors include any influence introduced by the human reader, e.g., lack of training or change of readers. By nature, they can be minimized but not eliminated entirely. Zange et al.^13^ analyzed the human influence in CMR and defined tolerance intervals as the intra-observer variabilities occurred in multiple measurements.

In an effort to identify the root cause of non-human potential confounding factors, dates of various events were collected. These dates included the introduction of new CMR readers, the relocation of the MR scanner, MR scanner software updates as well as quantification postprocessing software changes. Furthermore, a survey of all clinical and research studies conducted on the scanner have been collected, in order to detect any influence of an uneven distribution of indications.

### Phantom experiment—synthetic data generation

In order to provide a proof of concept, a phantom was created by simulating a bimodal normal variable as shown in Fig. 4a). The resulting samples are drift-free by definition, since there is no dependence on the time component (Fig. 4b). We drew 20,000 samples, which roughly represents the size of our present dataset. Figure 4c) is perceptually equal to Fig. 4b), however, here we added 5 ml to all LV SV values from x = 10,000 onwards. Computing MM for different window sizes gives Fig. 4b), the same computation on the shifted dataset gives Fig. 4c).

### Statistical method and distortion model

**Figure 1:**
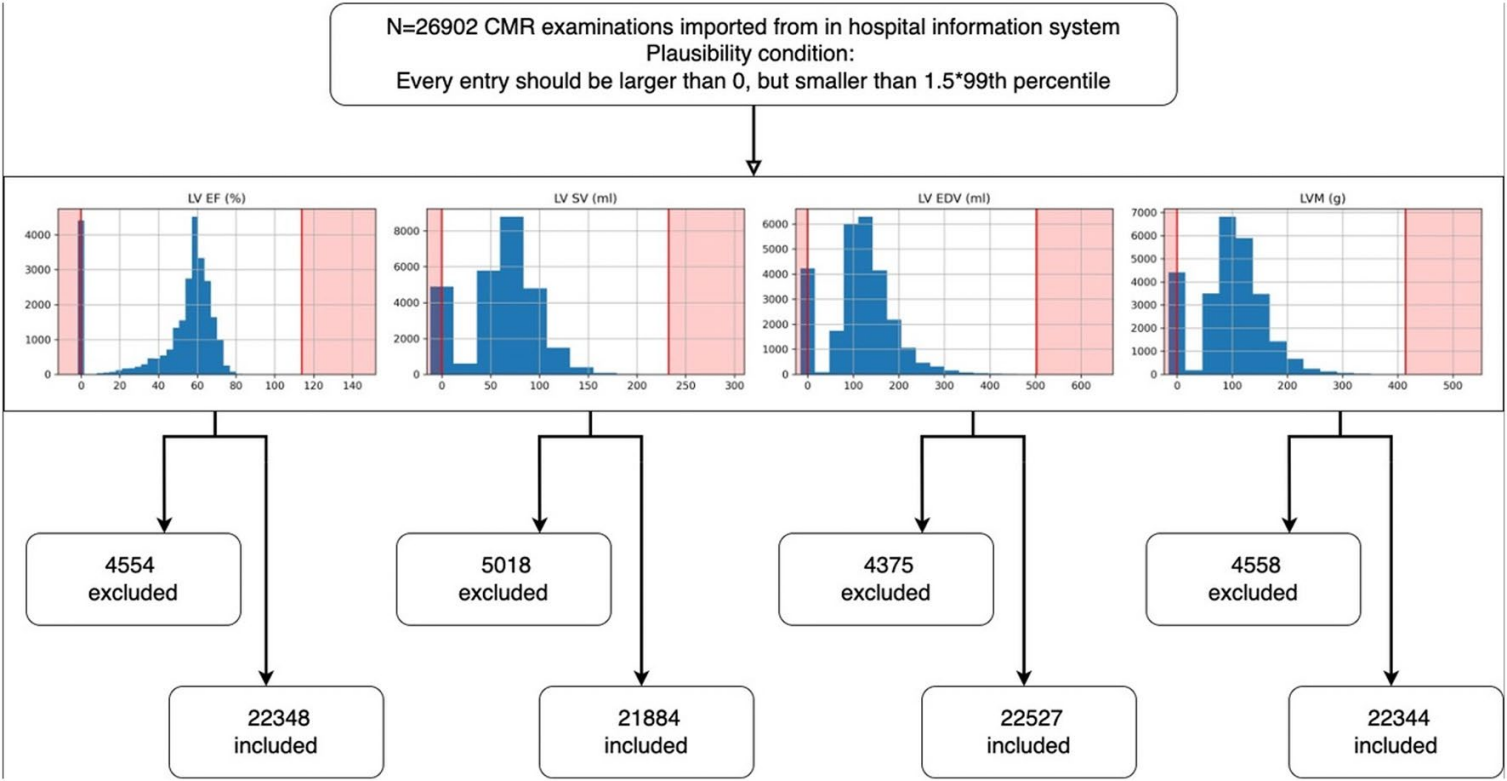
Flowchart of data selection. Abbreviations: CMR, cardiac magnetic resonance; LV EF, left ventricular ejection fraction; LV EDV, left ventricular end-diastolic volume; LV SV, left ventricular stroke volume; LVM, left ventricular mass.

Data sets were reviewed for suitability for every parameter individually. In order to be included in the analysis, a data point had to be strictly larger than zero and smaller than 1.5 times the 99th percentile of the uncleaned dataset. An overview of all data selection is included in Fig. 1. Furthermore, the date of examination had to be a valid date with an existing day, month and year.

The previously mentioned confounding factors are time-discrete in nature, meaning that the individual distorting event happened at a single timepoint. Therefore, the quantitative parameters will react with an immediate introduction of a bias which causes a drift of the quantitative parameters over time. The bias is the information we are looking for and is mathematically convoluted with the natural variations from patient to patient (the noise). It is therefore not easy to detect, especially when the extent of the bias is smaller than the statistical variation. The sought method needs to suppress the noise and augment the bias to the emergence (or the vanishing) of a confounding factor.

In this paper we chose to use moving median over time with a fixed window size. Therefore, the analysis will be influenced by the prescribed window size. Larger ones lead to stronger noise suppression but also longer time periods until an introduced bias becomes visible. Therefore, a trade-off must be made, i.e., maximizing the time between two false positive alerts (“Average Run Length”) on one hand side and minimizing the time to a true positive alert (“Average Delay Time”) for an underlying bias on the other side^22^.

In this paper we analyzed two different window sizes with 250 and 750 samples were analyzed, roughly reflecting a one month and one quarter period of scanning time. The window sizes are an estimate for a time-frame during which there is an even distribution of indications for CMR).

**Figure 2:**
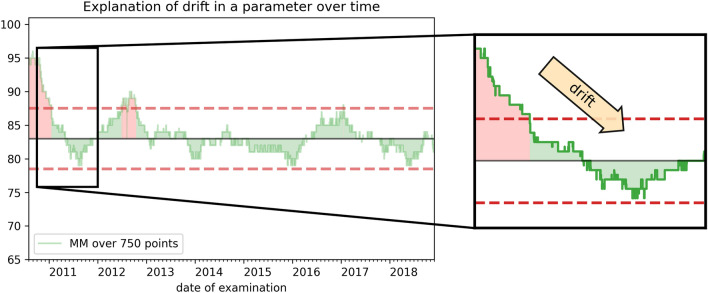
Quality control charts scheme explained by means of a moving median (MM) chart of left ventricular stroke volume (LV SV) values. The green curve shows the MM with a window of 750. The horizontal black line shows the median of all LV SV values. The red dashed lines show the tolerance interval boundaries. The area under the curve is colored green or red, to show that the curve is inside and outside of the tolerance interval respectively. A box is drawn into the plot, which transfers the reader to a zoom on the right side of the figure. The zoomed figure shows the drift inside the curve with an arrow.

### Statistical analysis

The moving median values are plotted in time-dependent charts with added upper and lower boundary limits (“control limits”)^23,24^.

These “control charts” are used to determine whether quantitative parameter results are within expectations (“in control”) or outside (“out of control”). Deviations will be handled as “alerts” and indicate potential confounding factors. As control limits, we used the tolerance intervals defined by Zange et. al.^13^.

**Table 1:** Demographical summary, giving the number of scans per parameter, as well as mean value with standard deviation and median with minimum and maximum values.

	Female (N = 8553)	Male (N = 11,784)	*P*-value
Age			
Mean (SD)	59.1 (16.3)	58.2 (16.0)	< 0.001
Median [Min, Max]	61.0 [7.00, 94.0]	60.0 [9.00, 94.0]	
Height (cm)			
Mean (SD)	164 (9.94)	177 (8.25)	< 0.001
Median [Min, Max]	164 [99.0, 760]	178 [86.0, 203]	
LV EF (%)			
Mean (SD)	60.9 (9.68)	56.9 (11.5)	< 0.001
Median [Min, Max]	62.0 [11.0, 98.0]	59.0 [7.00, 85.0]	
LV EDV (ml)			
Mean (SD)	127 (36.6)	171 (55.6)	< 0.001
Median [Min, Max]	121 [11.0, 457]	162 [11.0, 493]	
LV SV (ml)			
Mean (SD)	75.5 (18.1)	93.9 (25.5)	< 0.001
Median [Min, Max]	74.0 [15.0, 195]	92.0 [3.00, 231]	
LVM (g)			
Mean (SD)	104 (29.4)	152 (42.7)	< 0.001
Median [Min, Max]	99.0 [1.00, 406]	145 [6.00, 411]	

LV EF, left ventricular ejection fraction; LV EDV, left ventricular end-diastolic volume; LV SV, left ventricular stroke volume; LVM, left ventricular mass. The *P*-value indicates the result of the two-sample t-test on the respective rows.

A control chart is shown in Fig. 2. The control limits are depicted with dashed lines, the solid black line depicts the median value over the whole time period. It also shows the basic deviations we will focus on in this paper: Any entry of the moving median curve outside of the tolerance interval is regarded as an alert. The number of alerts is quantified by an aggregation to percentage per year.

**Figure 3:**
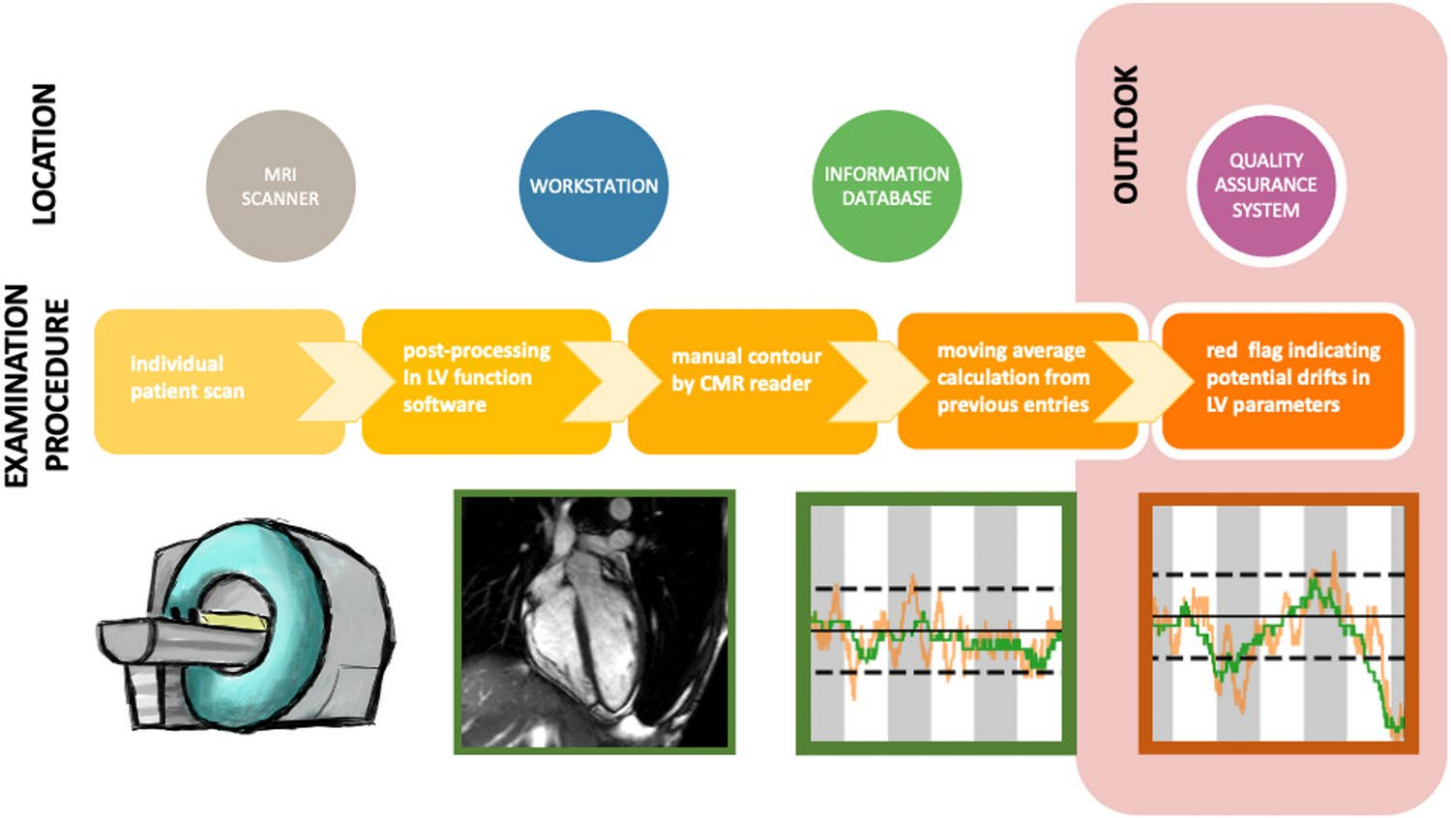
Flowchart of quality control procedure. The examination procedure can be divided into multiple steps with varying processing locations. All these steps may have an impact on the result of the examination. Abbreviations: CMR, cardiac magnetic resonance; LV, left ventricle.

CMR examinations were exported from the hospital information system as a comma-separated-values (CSV) file. This file was processed using Python 3.7^25^ on MacOS. The function used for computing moving medians is the “pandas”^26^ function “.rolling().median()”. For the generation of Table 1, we used Rstudio^27,28^ with the library “Table1”^29^. The statistical test used inside Table 1 is a two-sample t-test.

The whole process of the imaging chain and the downstream analysis is shown in Fig. 3.

For our proof of concept, we used the Granger Causality Test^30^. This is a statistical test, often used in econometrics, for testing causality between two time series.

## Results

### Phantom experiment—synthetic data

**Figure 4:**
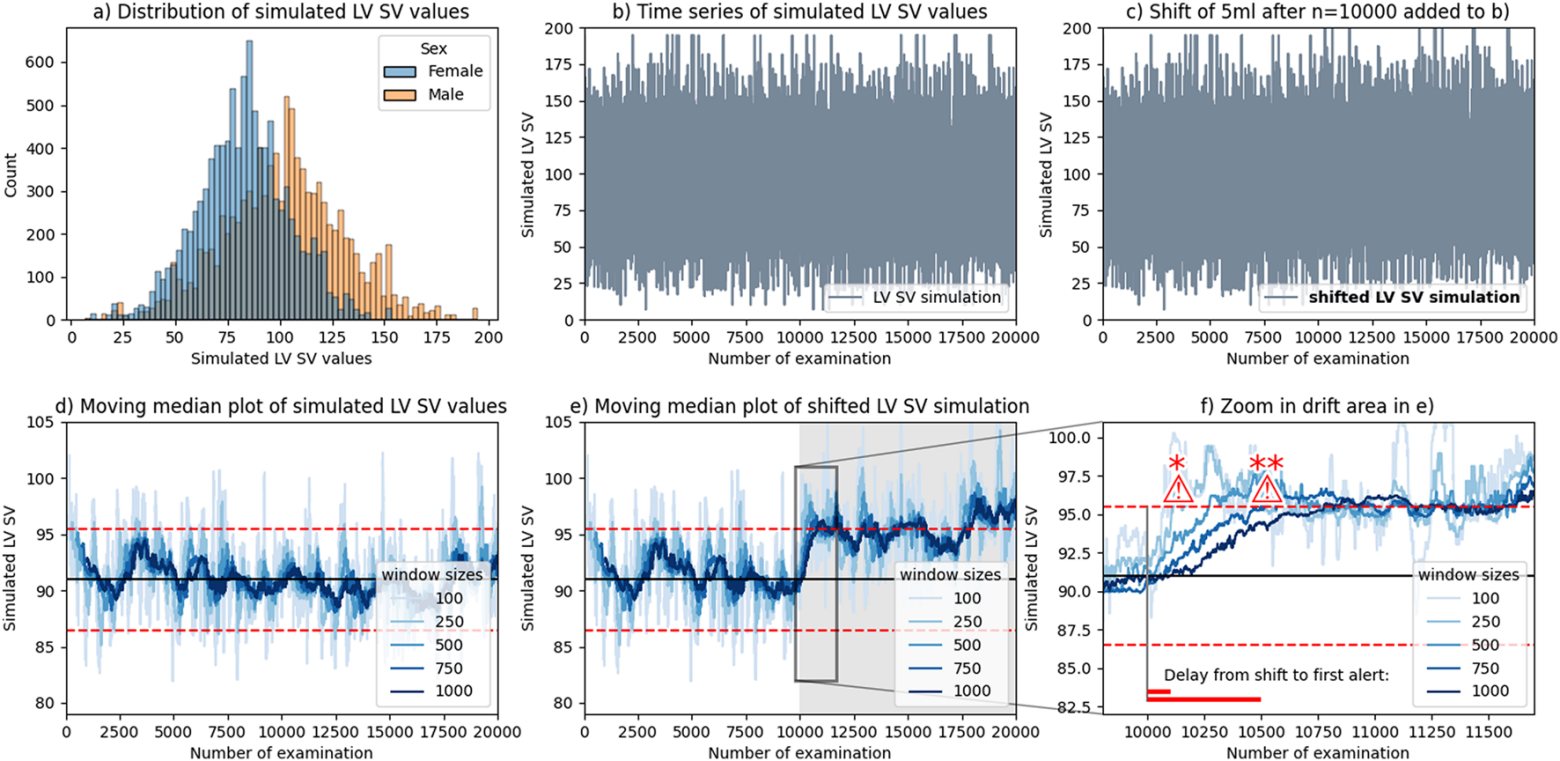
Simulated phantom dataset with a demonstration of the proposed method. (**a**) Bi-modal distribution of simulated LV SV values. (**b**) 20,000 random samples of the LV SV dataset were drawn and concatenated to simulate a stationary time series. (**c**) The data from (**b**), where 5 ml have been added to the last 10,000 samples to demonstrate a sudden and permanent change in the setup. No perceptual difference is visible. (**d**) The moving median of the data shown in (**b**) with different window sizes. Lines in different shades of blue represent aggregations of window sizes ranging from 100 to 1000. Red dashed lines indicate the tolerance interval (**e**) The moving median of the data shown in (**c**) with different window sizes. Lines in different shades of blue represent aggregations of window sizes ranging from 100 to 1000. Red dashed lines indicate the tolerance interval. The box with black borders indicates the area of drift. (**f**) Zoom in to the area of (**e**) containing the drift. The exclamation mark notes a potential alarm that could be issued by the system. Abbreviations: LV SV, left ventricular stroke volume; MM, moving median; * shows the alert of window 250; ** shows the alert for window size 750.

Our proof of concept demonstrates that a permanent shift in data magnitude will translate into a drift in the MM curves (Fig. 4d,e,f). The Granger Causality Test gave a significant (*P* < 0.0001) result for the confounder to have caused the dummy drift dataset. As a plausibility-check, we tested whether the dummy drift caused the confounder, which was not significant (*P* = 0.93).

### Demographics

Between March 18th 2010 and March 12th 2019, N = 26,902 CMR examinations were recorded in the clinical data management system of the Helios hospital in Berlin-Buch. Of N = 26,902 datasets, N = 20,337 datasets could be included for evaluation of all parameters (Table 1), after excluding unreasonable values as previously described in the methods section. The data spans 111 months, with an average of 197 patients (82 females and 115 males) per month. This yields to 10 years which could be included, with an average scan count of 2188 per year (912 females and 1276 males). Table 1 summarizes the number of scans that could be included for each parameter including mean, standard deviation and median values.

**Figure 5:**
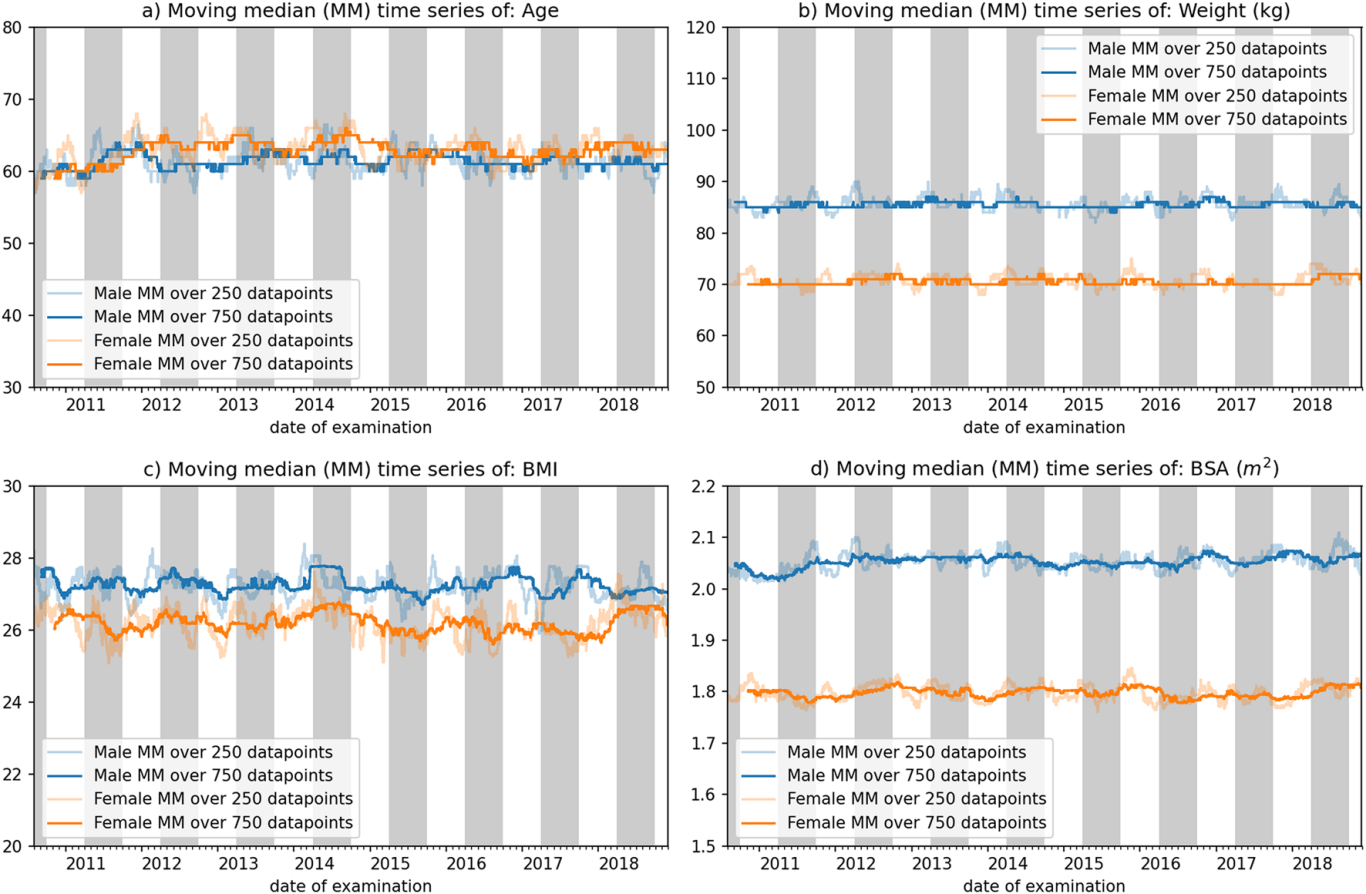
Moving median (MM) time series plots of age, weight, body-mass-index (BMI) and body-surface-area (BSA) (split by men and women) using windows of sizes 750 and 250. Gray and white background indicates time frames of staff rotation. The blue lines in every subplot represent the male population and the orange lines the female population. The faded lines represent a window size of 250 and the fully colored lines a window size of 750.

The time series curves of the moving median with window sizes 250 and 750 regarding age, weight, BMI and BSA are shown in Fig. 5. The data was divided by sex in order to capture potential differences. Over the course of the analysis, the age and weight of the populations remained stable with a slight but insignificant increase in age. We attributed this increase to the overall aging population. The weight curves as well as BMI showed the expected differences between the sexes, but were free from drifts.

**Figure 6:**
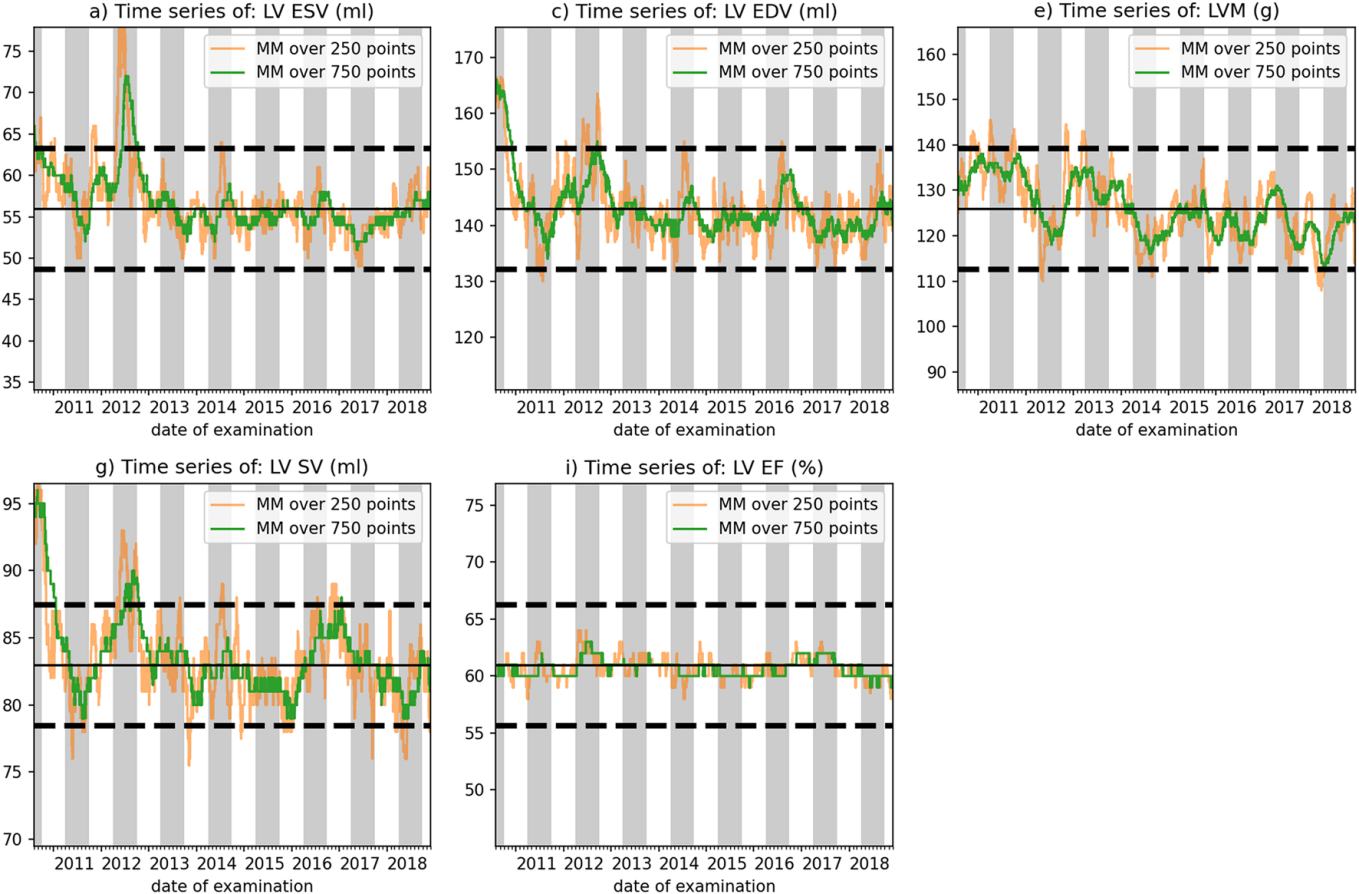
Time-dependent analysis of key parameters in relation to tolerance intervals. Tolerance intervals are marked as black dashed lines for each LV function parameter. Gray and white background indicates time frames of staff rotation. The orange line represents a window size of 250 and the green line a window size of 750. Abbreviations: MM, moving median; LV EF, left ventricular ejection fraction; LV ESV, left ventricular end-systolic volume (computed using LV EDV and LV SV); LV EDV, left ventricular end-diastolic volume; LV SV, left ventricular stroke volume; LVM, left ventricular mass.

The time-dependent charts of left ventricle parameters in relation to the control limits are shown in Fig. 6. It indicates several alerts and major drifts pointing to unwanted changes in the measurement process. The detailed analysis revealed different degrees of change of the analyzed parameters. The LV EF values stay well within the defined control limits, for both windows sizes 250 & 750 (Fig. 6e). The LV SV values (Fig. 6d) show a major drift from higher values in 2011, values most of the times within the control limits in 2012 and subsequent years with a general tendency to lower values. There are some short-term alerts where the mean values are below the lower control limit, e.g., mid 2018. These alerts occur using both window sizes, but as expected, less so using the window of size 750. Like LV SV, in the LV EDV (Fig. 6b) a large drift can be seen as well as the signal leaving the upper control limit in the early years, but less pronounced. In the later years, the signals stay within the limits, especially for the window of size 750, where from 2013 on, there were no fast changes or systematic drifts. We observe that the LV ESV curve follows a similar pattern as the LV EDV but displays different peak magnitudes. There is one peak, reaching outside of the tolerance boundaries, which lies entirely inside a gray shaded area (Fig. 6a). This indicates that the change happens at the same time as new staff changed into CMR rotation. The effect does not seem to be permanent throughout the 1-year duration of the rotation. Instead, the LV ESV values go back to baseline and remain there. The LVM (Fig. 6c) shows no large drift in the beginning, but short-term alerts especially in 2018 and again, less pronounced for the window of size 750. There is a general drift from higher values in the early years towards lower values in the later years albeit staying within the control limits. Table 2 show the counts of all these alerts for all parameters, separated between 2012 and 2013. The indexed LV ESV, LV EDV, LV SV and LVM are given in the Supplementary Information. Their curve progression is similar to the non-indexed respective curves. However, the magnitude is smaller and the drifts also seem less pronounced.

**Table 2:** Mean percentage of MRI examinations per year of an LV parameter crossing the limits defined by tolerance interval.

LV parameter	Alert/Year in % Before 2013		Alert/Year in % After 2013	
Window size	250	750	250	750
ESV (computed)	25	16	0	0
EDV	32	30	4	0
LVM	14	0	4	0
EF	0	0	0	0
SV	37	44	14	4

LV, left ventricle; ESV*, end-systolic volume (computed using LV EDV and LV SV); EDV, end-diastolic volume; LVM, left ventricular mass; EF, ejection fraction; SV, stroke volume.

As a next step, we attempted to connect the identified potential confounding factors to the alerts. Those included one relocation of the scanner, a scanner upgrade, change of formulas in the analyzing software and the change of the physician team every 6 months. However, none of the drifts or alerts could be safely pinpointed to these factors.

Special attention was given to the LV EDV and LV SV curve progressions as they are similar in shape whereas the LVEF stays within the tolerance interval. As the LVEF is calculated with

$$LVEF= \frac{LV SV}{LV EDV}= \frac{LV EDV-LV ESV}{LV EDV}$$

it seems that the changes are cancelling each other out such that the LV EF stays within the limits. That led to the hypothesis that the formulas to calculate the LV EDV and LV ESV within the applied commercial analysis software were changed in a similar fashion. However, despite intensive investigation, it was not possible to offer a definite answer on this hypothesis in our retrospective setting.

In general, the window size of 750 suppresses fast statistical changes more than the 250 window and therefore produces fewer alerts (Table 2).

## Discussion

In this study, we were able to transfer established methods of systematic quality assurance from in-vitro diagnostics to routine imaging lab settings for the first time. Time series smoothing with moving medians and established tolerance ranges as control limits enabled the detection of alerts pointing to potential quality issues. They were found in different parameters whereas their number and extent depended on the chosen window size of the rolling median. In the last 6.5 years there were only 4% alerts per year and no major drifts visually identified. Within the first 2.5 years of the analyzed timeframe there were 44% alerts per year and 4 major drifts. Potentially, improved image quality due to technical developments produced more artifact-free images. In our retrospective study we are more interested in detecting the long-lasting confounding factors in general, rather than detecting them quickly after they occurred. As such, the window of size 750 seems to be more appropriate, because the noise is suppressed more compared to the window of size 250, hence avoiding false alerts. The longer delay time this choice implies is negligible due to the retrospective character of the study.

In general, the size of window needs to be chosen carefully and should reflect the individual intention of the quality assurance process. Short delay times would mandate smaller window sizes but will cause more false positives alerts, larger window sizes are to be preferred if delay times are less important than stable alerts. A computational approach for computing an optimized window size could be attempted using bias detection simulation^31^. In other quality control applications, the window sizes are computed from the maximum number of false positives^24^. We deviated from this conventional method for exploratory purposes here.

The LV EF curve never left the tolerance interval, despite both LV EDV and LV ESV showing drifts. Nevertheless, LV EF seems to have a greater resilience for large changes in LV EDV than LV SV when looking at the asymptotic behavior of the functions.

The LV ESV, based on calculated numbers, leaves the tolerance boundaries which constitutes an alert. The LV EDV and LV ESV deviations may have been caused by formula updates in different post-processing software versions. However, since we cannot access the exact formulas used for the LV parameters over separate software versions, this hypothesis remains elusive. Nonetheless, the deviations were not clinically relevant, as most of the clinical decisions are based on LV EF.

Having said that, this underlines the importance of conducting the described quality assurance in a prospective way as it would allow to immediately analyze the deviations when they occur, hence ruling out any potential clinically relevant impacts.

Consequently, these alerts triggered investigations as to the causes (confounding factors) of the measurements leaving the control limits. As typical reasons for quality impairment in a laboratory setting do not apply in an imaging setting, we analyzed imaging specific hypotheses of confounding factors. Specifically, we analyzed a relocation of the scanner, a scanner upgrade, change of formulas in the analyzing software and the change of the physician team every 6 months. Despite comprehensive analysis, only one potential confounding factor could be aligned to the alerts given in this study. Finding and removing the cause of perturbation is the main goal of any quality assurance. All steps within an imaging chain may influence the results (Fig. 3). It starts with the specific scanner and changes in sequences including applied parameters. After image acquisition, postprocessing software is used to extract clinical parameters which are then stored in a hospital information system. Application of different software and promoting different readers influence quantitative results^11,13^, but so do internal software change based on using different formulas and potential changes within product sequences or commercial software which are out of in-depth control. In our retrospective analysis the cause of the alerts remained unknown. Potentially, a mathematical model could be developed in the near future but is not available today.

In a prospective study, a more precise evaluation of further potential confounders could become feasible. Nevertheless, this study highlighted the existence of both undetected and unwanted changes in the measurement chain.

The reason for the difficulties in finding the root causes of deviations is presumably the fact that the effects are years in the past. As the principal potential of the method to alert to quality problems has been shown, several improvements are planned. A refinement of the alert criteria seems to be indicated, i.e., by using control limits related to the clinical relevance of the parameters, rather than the intra-observer-based control limits. Most importantly, the method will be used in a prospective way, so that the root causes can be analyzed without time delay and therefore can be detected with a higher probability and subsequently be mitigated.

## Conclusion

In this paper, we could show that the statistical quality control method is suitable to assess the quality of quantitative parameters derived from images. It could therefore be used as part of the aforementioned quality assurance process as it alerts to problems and may enable effective countermeasures.

## Limitation

In this single center analysis of 26,902 CMR scans, the data was analyzed retrospectively. This limited the potential to identify the root causes of the confounding factors which were correlated to the alerts given in this study. Nevertheless, it highlighted the existence of undetected and unwanted changes in the measurement chain which is valuable per se.

The lack of confounders highlights the need for documentation in general hospital information systems to include relevant information inside the database such as details on scanner- and software upgrades. This reflects the retrospective nature of the data. Nevertheless, translating quality control mechanisms from different settings into imaging labs could be shown to be feasible.

The window sizes pose a tradeoff between finding error sources (true positives) and the evading false positive alerts. Therefore, no specific recommendation on a fixed window size can be given.

## Supplementary Information

Supplementary Information.

## Data Availability

The data that supports the findings in this study is/was available in cooperation with Helios hospital Berlin-Buch but restrictions apply to its current availability; for this study it was used under a license and is not publicly available. However, data is available from the authors upon reasonable request and with the permission of Helios hospital Berlin-Buch.
