# Supplementary material for: Translating principles of quality control to cardiovascular magnetic resonance: assessing quantitative parameters of the left ventricle in a large cohort
Source: Sci Rep. 2023 Feb 7;13:2205. doi: 10.1038/s41598-023-29028-7 (PMC9905535; doi:10.1038/s41598-023-29028-7)
Supplement: Supplementary file 1 — Supplementary Information. [file 41598_2023_29028_MOESM1_ESM.pdf]

Supplementary Information:

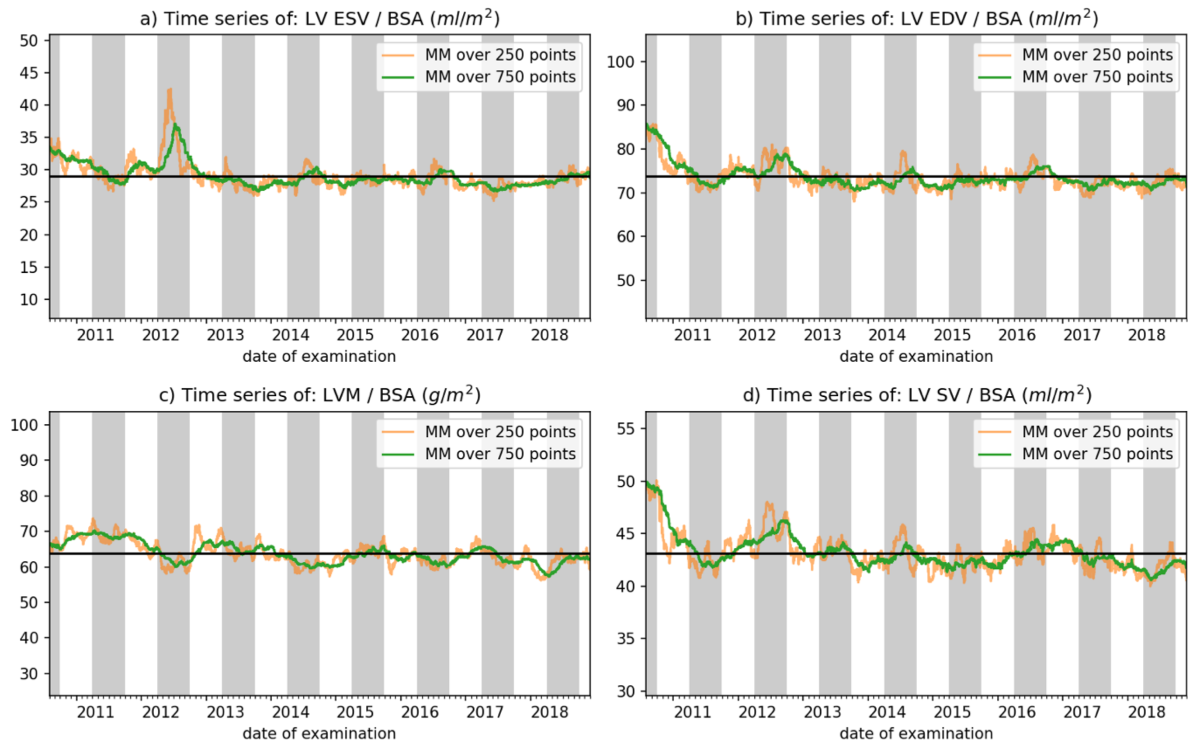

Figure S1: Time-dependent analysis of key parameters in relation to median value. Gray and white background indicates time frames of staff rotation. The orange and green lines represent window sizes of 250 and 750 respectively. Abbreviations: MM, moving median; LV ESV, left ventricular end-systolic volume (computed using LV EDV and LV SV); LV EDV, left ventricular end-diastolic volume; LV SV, left ventricular stroke volume; LVM, left ventricular mass; BSA, Body Surface Area.
